# Comparative study on the effect of human BST-2/Tetherin on HIV-1 release in cells of various species

**DOI:** 10.1186/1742-4690-6-53

**Published:** 2009-06-02

**Authors:** Kei Sato, Seiji P Yamamoto, Naoko Misawa, Takeshi Yoshida, Takayuki Miyazawa, Yoshio Koyanagi

**Affiliations:** 1 Laboratory of Viral Pathogenesis, Institute for Virus Research, Kyoto University, Kyoto, Kyoto 606-8507, Japan; 2 Department of Molecular and Cellular Biology, Graduate School of Biostudies, Kyoto University, Kyoto, Kyoto 606-8501, Japan; 3 Laboratory of Viral Pathogenesis, Center for Emerging Virus Research, Institute for Virus Research, Kyoto University, Kyoto, Kyoto 606-8507, Japan

## Abstract

In this study, we first demonstrate that endogenous hBST-2 is predominantly expressed on the plasma membrane of a human T cell line, MT-4 cells, and that Vpu-deficient HIV-1 was less efficiently released than wild-type HIV-1 from MT-4 cells. In addition, surface hBST-2 was rapidly down-regulated in wild-type but not Vpu-deficient HIV-1-infected cells. This is a direct insight showing that provirus-encoded Vpu has the potential to down-regulate endogenous hBST-2 from the surface of HIV-1-infected T cells. Corresponding to previous reports, the aforementioned findings suggested that hBST-2 has the potential to suppress the release of Vpu-deficient HIV-1. However, the molecular mechanism(s) for tethering HIV-1 particles by hBST-2 remains unclear, and we speculated about the requirement for cellular co-factor(s) to trigger or assist its tethering ability. To explore this possibility, we utilize several cell lines derived from various species including human, AGM, dog, cat, rabbit, pig, mink, potoroo, and quail. We found that ectopic hBST-2 was efficiently expressed on the surface of all analyzed cells, and its expression suppressed the release of viral particles in a dose-dependent manner. These findings suggest that hBST-2 can tether HIV-1 particles without the need of additional co-factor(s) that may be expressed exclusively in primates, and thus, hBST-2 can also exert its function in many cells derived from a broad range of species. Interestingly, the suppressive effect of hBST-2 on HIV-1 release in Vero cells was much less pronounced than in the other examined cells despite the augmented surface expression of ectopic hBST-2 on Vero cells. Taken together, our findings suggest the existence of certain cell types in which hBST-2 cannot efficiently exert its inhibitory effect on virus release. The cell type-specific effect of hBST-2 may be critical to elucidate the mechanism of BST-2-dependent suppression of virus release.

## Findings

To accomplish efficient release of HIV-1 particles, HIV-1 Vpu is required in certain cells (e.g., HeLa cells) but is dispensable in other cell types (e.g., HEK293 and Cos-7 cells) [1-3]. A previous report suggested that an inhibitory factor(s) for HIV-1 release is expressed in HeLa cells and the effect is attenuated by Vpu [4]. Recently, Neil and colleagues identified the inhibitor, hBST-2 (also called CD317 or HM1.24), in HeLa cells, and referred to this protein as "Tetherin" [5]. They also showed that the inhibitory action of hBST-2 on HIV-1 particle release was antagonized by Vpu, and they concluded that hBST-2 functions by tethering HIV-1 particles to the cell surface [5]. In addition, Van Damme and colleagues demonstrated that Vpu down-regulates hBST-2 from the surface of HeLa cells [6]. On the other hand, Miyagi and colleagues have recently reported that Vpu augments HIV-1 release without down-regulating surface hBST-2 in CEMx174 and H9 cells [7]. Therefore, the relevance of surface hBST-2 down-regulation and the antagonistic action of Vpu on the tethering ability of hBST-2 remain unclear.

**Figure 1:**
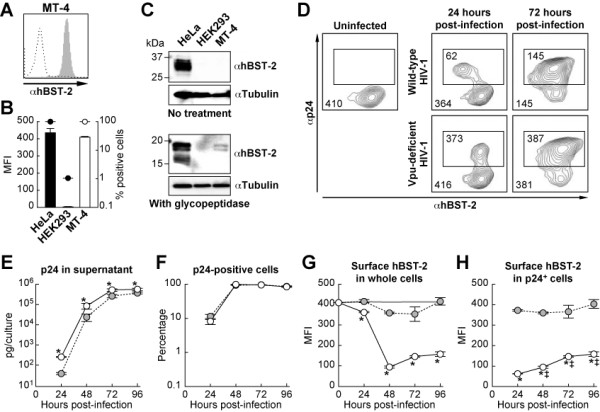
**Sequential analysis on the level of endogenous hBST-2 on the surface of HIV-1-infected human T cells**. (A and B) MT-4 cells were stained with a mouse anti-hBST-2 antibody, and the surface expression of endogenous hBST-2 (filled in gray) was analyzed by flow cytometry as described in the Materials and Methods. Isotype IgG was used as a negative control (broken line). A representative result (A) and summarized graph (B) are shown. The level of endogenous hBST-2 on the surface of MT-4 cells (opened bar and circle) is compared to that of HeLa and HEK293 cells (filled bars and circles). MFI is represented in bars (Y-axis on left), and the percentage of hBST-2-positive cells is represented in circles (Y-axis on right, log scale). (C) The level of endogenous hBST-2 expression in HeLa, HEK293, and MT-4 cells was analyzed by Western blotting (top panel). For clear detection of hBST-2, the cell lysates were treated with glycopeptidase as described in the Materials and Methods, and the level of deglycosylated hBST-2 was analysed by Western blotting (bottom panel). The input was standardized to Tubulin, and representative results are shown. kDa, kilodalton. (D-H) MT-4 cells were infected with either wild-type or Vpu-deficient HIV-1 (MOI 0.1). Endogenous hBST-2 on the cell surface and intracellular expression of p24 were sequentially analyzed by flow cytometry, and representative profiles are shown (D). The number in the corner of the plot indicates MFI of hBST-2 on the surface of whole cells, and that in the square in the plot indicates MFI of hBST-2 on the surface of p24-postive cells. The amount of p24 in the culture supernatant (E), the percentage of p24-positive cells (F), the level of hBST-2 on the surface of whole cells (G), and the level of hBST-2 on the surface of p24-positive cells (H) following infection with either wild-type (opened circles with line) or Vpu-deficient (filled circles in gray with broken line) HIV-1 were sequentially measured. The amount of p24 in the culture supernatant was quantified by p24 ELISA, and the other data were obtained by flow cytometry as described in the Materials and Methods. Gray line in panel G indicates MFI of surface hBST-2 on mock-infected cells. All experiments were performed in triplicate. Asterisks indicate statistical significance (Student's t test, P < 0.05) versus the values of Vpu-deficient HIV-1 at the same time point, and double daggers in panel H indicate statistical significance (Student's t test, P < 0.05) versus the values of wild-type HIV-1 at 24 hours post-infection. Error bars indicate standard deviations.

We first set out to analyze the level of endogenous hBST-2 expression in a T cell line (MT-4 cells) and compared this level to that found for adherent cell lines (HeLa and HEK293 cells). Although flow cytometry indicated that the level of surface hBST-2 on MT-4 cells was comparable to that expressed on HeLa cells, Western blotting indicated that the total amount of endogenous hBST-2 protein in HeLa cells was much more than the level found in MT-4 cells (Figures 1A–C). These results indicate that endogenous hBST-2 in MT-4 cells is predominantly expressed on the plasma membrane.

To analyze the sensitivity of endogenous hBST-2 on the surface of MT-4 cells to Vpu antagonism, MT-4 cells were infected with either wild-type or Vpu-deficient HIV-1, and the level of surface hBST-2 was subsequently monitored. The amount of released virions in the culture supernatant of wild-type HIV-1-infected cells was significantly higher when compared to that of Vpu-deleted HIV-1-infected cells (Figure 1E), while the percentage of p24-positive cells in wild-type HIV-1-infected culture was similar to that in Vpu-deleted HIV-1-infected culture (Figure 1F). These results suggest that the liberation of Vpu-deficient HIV-1 virions was impaired by endogenous hBST-2 in MT-4 cells. In addition, we clearly found that the surface expression of hBST-2 on wild-type but not Vpu-deleted HIV-1-infected cells (i.e., p24-positive cells) was severely down-regulated (Figures 1D and 1H). Although it has remained ambiguous in the literature whether endogenous hBST-2 on the surface of human T cells is down-regulated by HIV-1 infection [6,7], this is the first demonstration of the significant down-regulation of endogenous hBST-2 in T cells by Vpu which resulted from HIV-1 infection and not from transfection with a Vpu-expressing plasmid [6,8].

Following the rapid down-regulation of surface hBST-2 by infection with wild-type HIV-1, the surface expression of hBST-2 was gradually but significantly replenished along with HIV-1 expansion (Figures 1D and 1H). It is unclear how and why the surface levels of hBST-2 increased; however, our finding indicates that the level of down-regulation of surface hBST-2 on HIV-1-infected T cells would vary depending on the time after infection.

**Figure 2:**
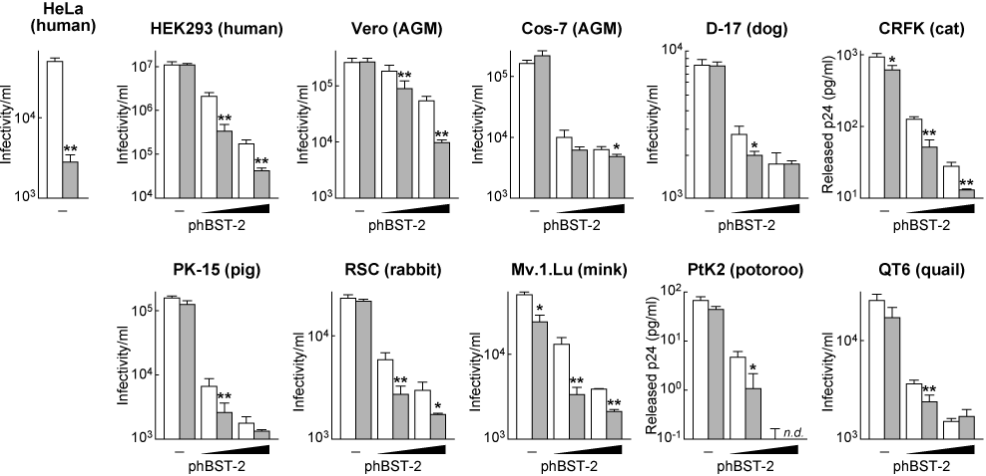
**Suppression of HIV-1 release by exogenous hBST-2 in various cell lines**. One microgram of pNL4-3 and pNL43-Udel was each co-transfected with (20 or 100 ng) or without (-) phBST-2 into several lines of cells as described in the Materials and Methods. The amount of wild-type (opened bars) or Vpu-deficient HIV-1 virion (bars filled in gray) released from HeLa, HEK293, Vero, Cos-7, D-17, PK-15, RSC, Mv.1.Lu, and QT6 was quantified by using TZM-bl cells, and the amount of HIV-1 released from CRFK and PtK2 cells was quantified by p24 ELISA. All experiments were performed in triplicate. Statistical significance (Student's *t *test) versus wild-type HIV-1 values is represented as follows: *, *P *< 0.05; **, *P *< 0.01. Error bars indicate standard deviations. *n.d*., not detectable.

Consistent with previous reports, our findings suggested that hBST-2 has the potential to attenuate HIV-1 release [5,6]. However, how hBST-2 acts against the release of HIV-1 particles remains unclear, and it is not known whether the hBST-2 function involves additional cellular co-factor(s). Since the potential of hBST-2 for the suppression of HIV-1 release has been reported only in primate cell lines [5-7], we hypothesized that hBST-2 may utilize co-factor(s) expressed uniquely in primate cells to tether virions. To investigate the role of hBST-2, we set forward to use various cell lines derived from 9 animal species including human, AGM, dog, cat, rabbit, pig, mink, potoroo, and quail. These cells were transfected with either wild-type or Vpu-deficient HIV-1-producing plasmid (pNL4-3 or pNL43-Udel). The amounts of released virions from HEK293, Vero, Cos-7, D-17, PK-15, RSC, Mv.1.Lu, and QT6 cells were quantified by TZM-bl titration assay [9], while those from CRFK and PtK2 cells were quantified by p24 ELISA because of their lower infectivity [10] (Figure 2). As previously described [4-6,11], HeLa cells were incompetent for the release of Vpu-deficient HIV-1 (Figure 2). In contrast, the other cell lines examined here were able to produce almost comparable amounts of Vpu-deficient HIV-1 when compared to the release of wild-type HIV-1 (Figure 2). These results indicate the absence in these examined cells of intrinsic factors which have the potential to be similar to hBST-2 and can be antagonized by Vpu.

Previous studies have shown that rhTRIM5α, a well-known restriction factor for HIV-1 replication [12,13], is able to efficiently elicit its suppressive ability for HIV-1 replication in feline CRFK cells, but not in canine D-17 cells [14,15]. These results suggest the species-specific ability of rhTRIM5α to suppress HIV-1 replication. To investigate the species-specific tethering ability of hBST-2, we next co-transfected an hBST-2-expressing plasmid (phBST-2) with either pNL4-3 or pNL43-Udel in the above examined cell lines and harvested released virions at 24 hours post-transfection. As shown in Figure 2, exogenous hBST-2 in these cell lines clearly suppressed the release of Vpu-deficient HIV-1 in a dose-dependent manner. This result strongly indicates that hBST-2 can tether released HIV-1 particles without any other unidentified co-factors that are expressed exclusively in primates. It remains conceivable that hBST-2 could employ certain elements ubiquitously expressed in many species for the tethering of released virions. Although it has been controversial whether wild-type HIV-1 release can be suppressed by ectopic hBST-2 or not [5,6], we observed here that the release of wild-type HIV-1 was attenuated by hBST-2 and that the efficiency of hBST-2 for the release of wild-type HIV-1 was significantly lower than that for the release of Vpu-deleted HIV-1 (Figure 2).

**Figure 3:**
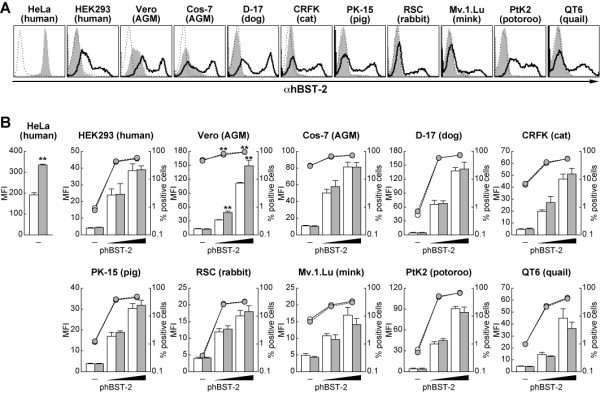
**Surface expression of exogenous hBST-2 in various cell lines**. (A) HEK293, Vero, Cos-7, D-17, CRFK, PK-15, RSC, Mv.1.Lu, PtK2, and QT6 cells were transiently transfected with 100 ng of phBST-2. phBST-2-transfected cells (black line) and mock-transfected cells (filled in gray) as well as HeLa cells (filled in gray) were stained with a mouse anti-hBST-2 monoclonal antibody, and the surface expression of hBST-2 was analyzed by flow cytometry as described in the Materials and Methods. Isotype IgG was used as a negative control (broken line). A representative result is shown. (B) One microgram of pNL4-3 and pNL43-Udel was each co-transfected with (20 or 100 ng) or without (-) phBST-2 into several lines of cells as described in Figure 2. The surface expression of hBST-2 on pNL4-3-co-transfected (opened bars and circles) and pNL43-Udel-co-transfected (gray bars and circles) cells was analyzed by flow cytometry. MFI is represented in bars (Y-axis on left), and the percentage of hBST-2-positive cells is represented in circles (Y-axis on right, log scale). All experiments were performed in triplicate. Statistical significance (Student's *t *test) versus wild-type HIV-1 values is represented as follows: *, *P *< 0.05; **, *P *< 0.01. Error bars indicate standard deviations.

Ectopically expressed hBST-2 was detected on the surface of all cell lines used in this study (Figure 3A). Unexpectedly, we found the staining with this antibody in native AGM cell lines, Vero and Cos-7 cells (Figure 3A) that increased in intensity when treated with IFN-α (data not shown). It is known that hBST-2 expression is induced upon IFN-α treatment in HEK293 cells [5,6]. Therefore, the antibody-specific staining and its increased signal intensity that we observed in the AGM cells could be due to the cross-reactivity of the anti-BST-2 antibody with endogenous AGM BST-2.

As previously reported [6], we also found that endogenous hBST-2 on HeLa cells was significantly down-regulated by transfection with pNL4-3, but not with pNL43-Udel (Figure 3B). In contrast, at 24 hours post-transfection, the down-regulation of exogenous hBST-2 on the surface of the other cell lines was hardly observed except for Vero cells (Figure 3B). However, after 48 hours post-transfection, we could detect significant down-regulation of ectopically expressed hBST-2 on the surface of cells co-transfected with either pNL4-3 or a Vpu-expressing plasmid [8] (data not shown). These results suggest that the level of Vpu expression at 24 hours post-transfection is sufficient to antagonize the tethering ability of hBST-2, while not down-regulating surface hBST-2. In support of our data, a recent report showed that Vpu enhances HIV-1 release in the absence of surface down-regulation of hBST-2 [7]. Taken together, these results indicate that the down-regulation of surface hBST-2 may be dispensable for the antagonism of tethering ability of hBST-2 by Vpu.

**Figure 4:**
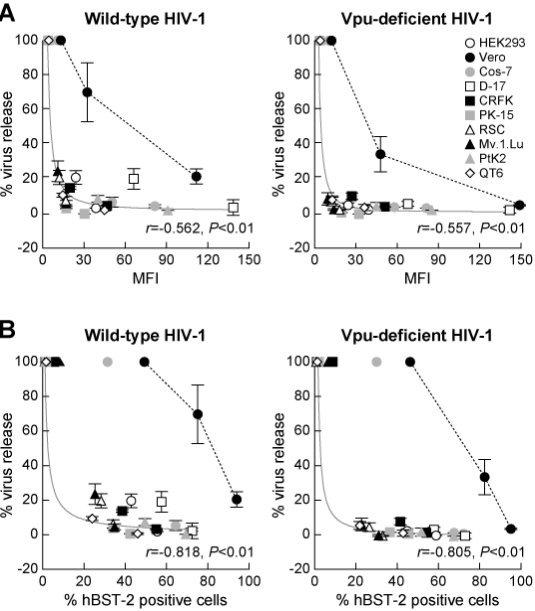
**Comparison of the level of exogenous hBST-2 on plasma membrane with its inhibition efficiency for HIV-1 release in various cell lines**. (A and B) The results shown in Figures 2 and 3 were summarized and rearranged as follows: the level of surface expression of hBST-2 is shown in MFI (A) and the percentage of surface hBST-2 positive cells (B) in the X-axis. To calculate % virus release (Y-axis), the infectivity of the culture supernatant of phBST-2-untransfected cells (for HEK293, Vero, Cos-7, D-17, RSC, Mv.1.Lu, and QT6 cells) or the amount of p24 in the culture supernatant of phBST-2-untransfected cells (for CRFK and PtK2) was defined as 100%. Statistical significance of the correlation between the level of surface hBST-2 (X-axis, shown in MFI or % positive cells) and % virus release (Y-axis) in the results from the 9 analyzed cells (HEK293, Cos-7, D-17, CRFK, PK-15, RSC, Mv.1.Lu, PtK2, and QT6 cells) was determined by Pearson's correlation test, and *P *< 0.01 was considered significant. Approximation curve of the result from the 9 analyzed cells is drawn in gray lines, and a representative result from Vero cells is drawn in broken line. *r*, Pearson's correlation coefficient.

We further assessed the results obtained from all the examined cell lines and focused on the correlation between the efficiency of particle release and the level of surface hBST-2 in these cells. All of the examined cell lines except for Vero cells showed significant suppression of virus release by exogenously expressed hBST-2 (Figure 4). In addition, a direct correlation between the suppression efficiency for virus release by hBST-2 and the level of surface hBST-2 was found in these cells with high correlation coefficients (Figure 4) and statistical significance (*P *< 0.01). On the other hand, the suppression efficiency for virus release by hBST-2 in Vero cells was relatively milder than in the other 9 cell lines even though Vero cells exhibited the highest levels of hBST-2 cell surface expression (Figure 4). Moreover, the result from Vero cells displayed a statistically different pattern than in the other cells (Figure 4, *P *< 0.01 by repeated measure ANOVA). These findings suggest that ectopic hBST-2 is unable to efficiently exert its inhibitory effect on virus release in Vero cells. One plausible explanation for this anomaly may be attributed to a defective IFN-α response. Although a previous study showed that the release of Vpu-deficient HIV-1 was suppressed upon IFN-α treatment [11], Vero cells are known to be genetically deficient in type I IFN genes, including IFN-α [16,17]. Therefore, it is conceivable that a signal cascade mediated by IFN-α may be needed to assist the tethering action of ectopic hBST-2, but that this cascade may not be operative in Vero cells because of its defects in type I IFN genes. Further studies in Vero cells will be needed to shed light on the unexplained aspects of the mechanism of suppression of virus release mediated by hBST-2.

It has recently been reported that hBST-2 has the potential to suppress the release of not only HIV-1 but also other retroviruses [18], Ebola virus [18], Lassa virus [19], and Marburg virus [18,19]. Therefore, further studies on the mechanism of BST-2 function will provide beneficial information leading to novel therapeutic strategies against several virus-induced diseases including AIDS.

## Methods

### Cell culture

HEK293 cells (human kidney), Vero cells (AGM kidney), Cos-7 cells (AGM kidney), rabbit skin cells (RSC, kindly provided by Dr. B. Roizman), and TZM-bl cells (obtained from AIDS reagent program, National Institute of Health) were maintained in low-glucose DMEM (Nikken) containing 10% FCS and antibiotics. D-17 cells (canine osteosarcoma), CRFK cells (feline kidney), PK-15 cells (porcine kidney), Mv.1.Lu cells (*Mustela vison*, mink lung), and QT6 cells (*Coturnix coturnix japonica*, quail fibrosarcoma) were maintained in high-glucose DMEM (Sigma) containing 10% FCS, 2 mM GlutaMax (Invitrogen), and antibiotics. PtK2 cells (potoroo kidney) were maintained in Eagle's minimum essential medium (Sigma) supplemented with 1 mM sodium pyruvate, 2 mM GlutaMax, 10% FCS and antibiotics. MT-4 cells were maintained in RPMI1640 (Nikken) containing 10% FCS and antibiotics. Mv.1.Lu cells and QT6 cells were kindly donated by Dr. A. Koito.

### Plasmid construction

To construct phBST-2, a *bst-2 *cDNA (GenBank: NM_004335, bases 10-552) was amplified by polymerase chain reaction from a human leukocyte cDNA library (Invitrogen), and the resulting fragment was inserted into peGFP-C1 (Clontech). Sequence of the construct was confirmed with an ABI 3130xl genetic analyzer (Applied Biosystems).

### Transfection and virus preparation

Cells were seeded in 6-well plate to appropriate densities 1-day prior to transfection and were transfected by using Lipofectamine 2000 reagent (Invitrogen) according to the manufacture's protocol. Briefly, 1 μg of pNL4-3 [20] or pNL43-Udel (kindly donated by Dr. K. Strebel) [1] was cotransfected with 20 or 100 ng of phBST-2. The amount of plasmid DNA for transfection was normalized to 2 μg per well. Four hour after transfection, culture medium was replaced freshly. The culture supernatant was harvested, centrifuged, and then filtrated with 0.45-μm filter (Millipore) to produce virus solutions at 24 hours post-transfection. All experiments were performed in triplicate. To prepare wild-type or Vpu-deficient HIV-1 for its infection assay, pNL4-3 or pNL43-Udel was transfected into HEK293 cells by the calcium phosphate method as previously described [21]. The prepared viruses were titrated by using peripheral blood mononuclear cells, and the TCID_50 _was calculated as previously described [22].

### TZM-bl assay

Quantification of the amount of released HIV-1 virion was performed by using TZM-bl cells as previously described [5]. Briefly, appropriate virus solution was inoculated into 1 × 10^5 ^TZM-bl cells per 12-well plate. The cells were harvested at 48 hours post-infection, and β-galactosidase assay was performed by using Galacto-Star Mammalian Reporter Gene Assay System (Applied Biosystems) according to the manufacture's procedure. Activity was measured with a 1420 ALBOSX multilabel counter (Perkin Elmer).

### p24 ELISA

The amount of HIV-1 virion released from CRFK, PtK2, and MT-4 cells was quantified by using HIV-1 p24 ELISA kit (ZeptoMetrix) according to the manufacture's instructions.

### Flow cytometry

Flow cytometry was performed as previously described [21]. A mouse anti-hBST-2 monoclonal antibody (donated by Chugai Pharmaceutical Co., Japan) [6,23] and a Cy5-conjugated donkey anti-mouse IgG antisera (Chemicon) were used. For costaining of cell surface hBST-2 and intracellular p24, the anti-hBST-2 monoclonal antibody was pre-labelled with Zenon Alexa Fluor 647 mouse IgG2a labelling kit (Invitrogen) according to the manufacture's protocol. Cell surface hBST-2 was stained with the pre-labelled anti-hBST-2 antibody, and the cells were permeabiliezed and fixed with BD Cytoperm/Cytofix solution (BD Pharmingen). Then, intracellular p24 was stained with a FITC-conjugated anti-HIV-1 p24 antibody (clone 2C2, kindly provided by Dr. Y. Tanaka) [24].

### Western blotting

Western blotting was performed as previously described [21] with some modification. Briefly, the cells were lysed with lysis buffer (1% NP-40, 50 mM Tris-HCl [pH7.5], 150 mM NaCl, 1 mM EDTA, 1 mM Na_3_VO_4_, and 1 mM PMSF). The lysates were separated by SDS-PAGE and transferred to Immobilon transfer membrane (Millipore). For detection, the mouse anti-hBST-2 monoclonal antibody, a mouse anti-Tubulin monoclonal antibody (clone DM1A; Sigma), and an HRP-conjugated horse anti-mouse IgG antibody (Cell Signalling) were used. It has been reported that hBST-2 is a highly glycosylated protein [25]. To remove the sugar chains in hBST-2 protein and detect hBST-2 more clearly, the lysates were treated with glycopeptidase F (TaKaRa) according to the manufacture's procedure.

### Statistical analyses

Student's *t *test was used to determine statistical significance, and *P *< 0.05 and *P *< 0.01 were considered significant. The Pearson correlation coefficient was applied to determine statistical significance for the correlation between the suppression efficiency for particle release by hBST-2 and the level of surface hBST-2 in the 9 kinds of cells lines (Figure 4), and *P *< 0.01 was considered significant. Repeated measure ANOVA was applied to determine statistical significance between Vero cells and the other cell lines (Figure 4), and *P *< 0.01 was considered significant.

## Abbreviations

h: human; BST-2: bone marrow stromal cell antigen-2; HIV-1: human immunodeficiency virus type 1; Vpu: viral protein U; AGM: African green monkey; ELISA: enzyme-linked immunosorbent assay; rhTRIM5α: rhesus macaque tripartite motif-containing 5 isoform α; phBST-2: hBST-2-expressing plasmid; IFN: interferon; AIDS: acquired immunodeficiency syndrome; DMEM: Dulbecco's modified Eagle medium; FCS: fatal calf serum; TCID_50_: 50% tissue culture infectious dose; FITC: fluorescein isothiocyanate; EDTA: ethylenediaminetetraacetic acid; PMSF: phenylmethylsulfonyl fluoride; SDS-PAGE: sodium dodecyl sulfate-polyacrylamide gel electrophoresis; HRP: horseradish peroxidase; MOI: multiplicity of infection; MFI: mean fluorescence intensity.

## Competing interests

The authors declare that they have no competing interests.

## Authors' contributions

KS and YK designed the research; KS, SPY, NM, TM, and TY prepared the materials; KS, SPY, and NM performed the experiments and analyzed the obtained data; KS and SPY prepared the figures; KS, TM, and YK wrote the manuscript.
